# GC-MS-based metabolomics approach to diagnose depression in hepatitis B virus-infected patients with middle or old age

**DOI:** 10.18632/aging.101535

**Published:** 2018-09-02

**Authors:** Lijuan Hou, Xiaoxia Wei, Ya Zhuo, Lei Qin, Fang Yang, Lili Zhang, Xinwen Song

**Affiliations:** 1 Department of Infectious Disease, The First Affiliated Hospital of Xinxiang Medical University, Weihui 453100, Henan, China; 2 Department of Gastroenterology, The First Affiliated Hospital of Xinxiang Medical University, Weihui 453100, Henan, China

**Keywords:** depression, hepatitis B virus, metabolomic, biomarker

## Abstract

Depression is concomitantly presented in hepatitis B virus (HBV)-infected patients (HB). However, there is still no objective method to diagnose HBV-infected patients with depression (dHB). Therefore, in this study, a gas chromatography-mass spectrometry (GC-MS)-based metabolomic approach was employed to profile urine samples from 59 dHB and 52 HB (the training set) in order to identify urinary metabolite biomarkers for dHB. Then, 41 dHB and 35 HB (the testing set) were used to independently validate the diagnostic generalizability of these biomarkers. In total, 13 differential metabolites responsible for the discrimination between dHB and HB were identified. These differential urinary metabolites belonged mainly to Lipid metabolism and Amino acid metabolism. A panel consisting of six urinary metabolite biomarkers (ethanolamine, azelaic acid, histidine, threitol, 2,4-dihydroxypyrimidine and levulinic acid) was identified. This panel was capable of distinguishing dHB from HB with an area under the receiver operating characteristic curve (AUC) of 0.986 in the training set. Moreover, this panel could classify blinded samples from the testing set with an AUC of 0.933. These findings indicated that the GC-MS-based metabolomics approach could be a useful tool in the clinical diagnosis of dHB, and the identified biomarkers were helpful for future developing an objective diagnostic method for dHB.

## Introduction

Although preventable with safe and effective vaccines, hepatitis B virus (HBV) infection is still a serious global health concern [1]. It is estimated that there are 400 million people with chronic hepatitis B worldwide, and thousands of patients succumb annually to end-stage liver cirrhosis and hepatocellular carcinoma [2]. Up to now, the available treatment methods could not completely eliminate the HBV from the body. The incurable nature of this disease often causes many negative emotions, such as depression and anxiety disorder. Moreover, the misunderstanding of the infectivity of HBV could increase the psychological pressure on HBV-infected patients, thus promoting the development of mental disorders, such as depression [3].

Depression is a seriously debilitating mental disorder. The previous study reported that it affected up to 200 million people (approximately 3% of the world's population) in 2015 [4]. It could affect a person's behavior, thoughts and feelings [5,6]. Gallegos-Orozco et al. reported that depression was prevalent in chronic HBV-infected patients [7]. Depression could decrease the quality of life and social activities in these patients, and it is also linked to a worse outcome in multiple medical disorders including viral illnesses [8]. What's more, depression could impinge on self-management ability and then reduce the patients compliance with prolonged therapeutic regimens [9]. However, the prevention and treatment of depression is often overlooked in HBV-infected patients.

Currently, the pathogenesis of depression is still unclear [10-12]. The psychiatrists still rely on the subjective identification of symptomatic clusters to diagnose depression. But, due to the highly heterogeneous clinical presentation of depression, this method results in a considerable error rate [13]. Recently, metabolomics has been increasingly used to identify potential biomarkers for neuropsychiatric disorders [14,15]. In our previous studies, using the nuclear magnetic resonance (NMR) spectroscopy-based metabolomic approach, we firstly observed the divergent urinary metabolic phenotypes between HBV-infected patients with depression (dHB) and HBV-infected patients without depression (HB), and successfully identified several potential biomarkers for dHB [16,17]. However, considering that no single analytical technology could provide adequate coverage of the entire human metabolome in any biological samples [18,19], it is important to use the complementary metabolomic platforms to identify novel biomarkers for dHB. Some studies have proved the especially valuable of this complementation for psychiatric disorders, such as BD and schizophrenia [14,20]. Therefore, in this global metabolite profiling study, a gas chromatography-mass spectrometry (GC-MS)-based metabolomic platform was used to further study the divergent urinary metabolic phenotypes between dHB and HB, and identify some novel urinary metabolite biomarkers for future development of a urine-based diagnostic test for depression in HBV-infected patients.

## RESULTS

### Baseline data

**Figure 1:**
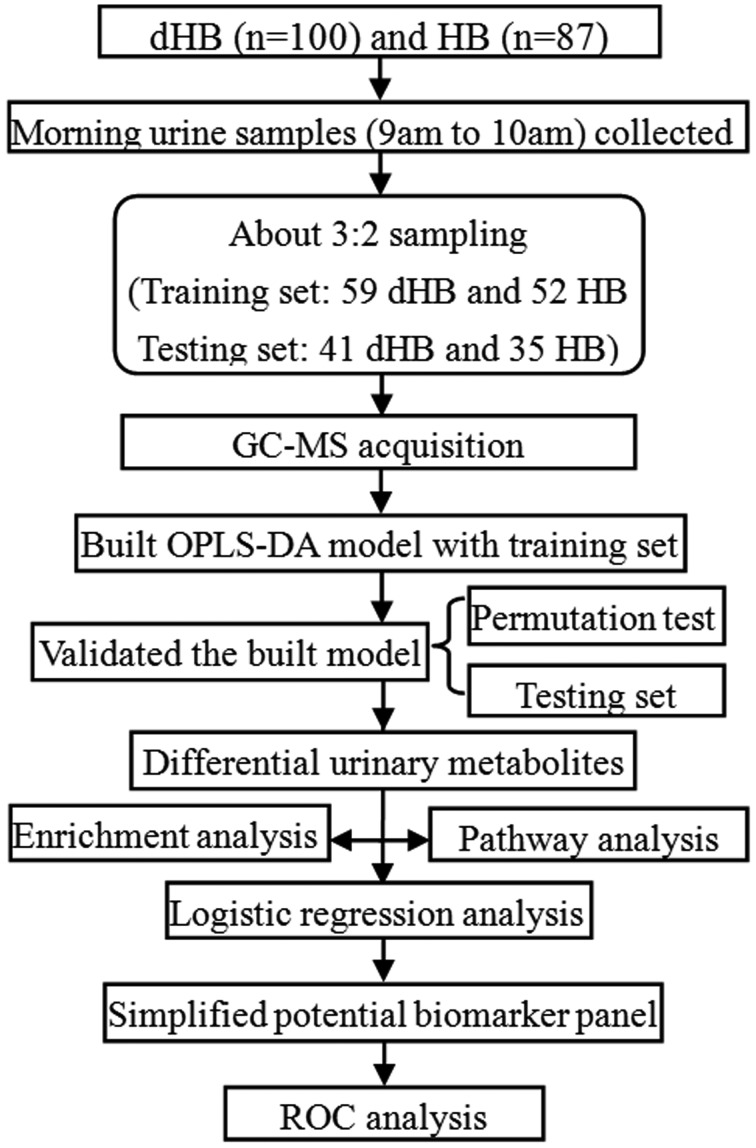
Flow chart of the study strategy in this work.

In total, 87 HB and 100 demographically matched dHB were included in this study. There were 11 patients in the HB group and 16 patients in the dHB group receiving medicines for treating HBV, such as telbivudine, lamivudine, interferon and adefovir dipivoxil. According to the results of our previous metabolomic studies [16,17], the statistical power could be up to 0.83 when the dHB group had 100 patients and HB group had 87 patients. Using the independent samples to validate the diagnostic performance of the identified panel was essential before it could be used clinically. Thus, the included patients were divided into training set and testing set. Finally, there were 52 HB (28 female and 24 male) and 59 dHB (28 female and 31 male) in the training set, and 35 HB (16 female and 19 male) and 41 dHB (23 female and 18 male) in the testing set. The training set was used to identify the biomarker panel, and the testing set was used to independently validate the diagnostic performance of the obtained panel. There were no significant differences in age, gender, or body mass index (BMI) between the two groups in both sets. The detailed information was described in Table 1. The flow chart of the study strategy was described in Figure 1.

**Table 1:** Demographic and clinical characteristics of the included patients.

	**Training set**			**Testing set**		
	HB	dHB	P-value	HB	dHB	
Sample Size	52	59	-	35	41	
Age (years)^a^	45.7±14.5	45.0±17.1	0.82	45.6±15.2	50.6±16.1	0.17
Sex(F/M)^b^	28/24	28/31	0.50	16/19	23/18	0.37
HDRS^a^	1.7±1.0	23.9±3.9	<0.00001	1.4±1.2	23.4±3.7	<0.00001
BMI^a^	22.8±3.8	22.5±3.7	0.67	21.7±3.9	21.8±3.4	0.93

^a^Mean ±standard deviation, p-value was from student's t-test. ^b^ p-value was from Chi-square test. HB: hepatitis B virus infected patients without depression; dHB: hepatitis B virus infected patients with depression; F: female; M: male; BMI: Body Mass Index; HDRS: Hamilton depression rating scale.

### OPLS-DA model

**Figure 2:**
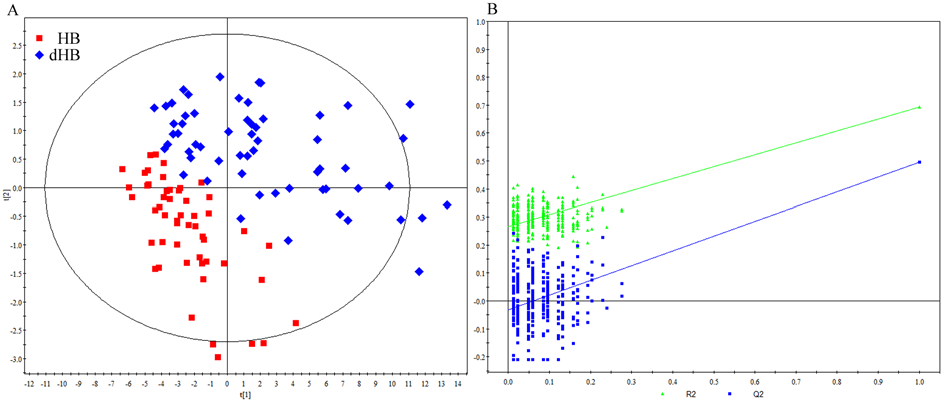
**Metabonomic analysis of urine samples from HB and dHB.** (**A**) OPLS-DA score plots showing an obvious separation between dHB (blue diamond) and HB (red square) in the training set; (**B**) 300-iteration permutation test showing the corresponding permuted values (bottom left) as significantly lower than original R^2^ and Q^2^ values (top right), demonstrating the OPLS-DA model's robustness.

**Figure 3:**
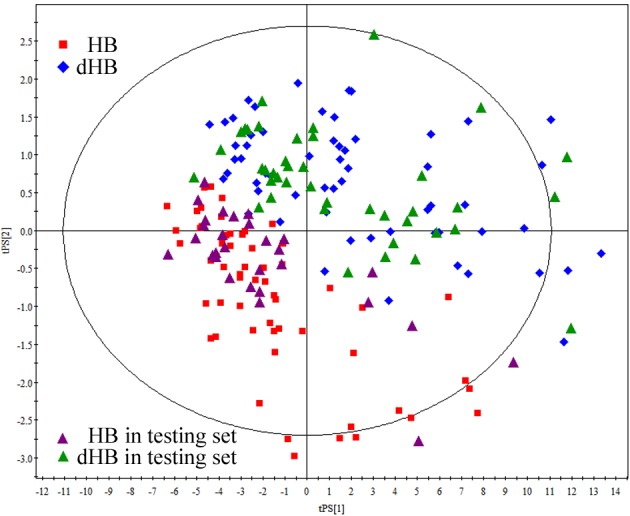
**T-Predicted scatter plot from the OPLS-DA model built with HB (red square) and dHB (blue diamond) in the training set.** The 31 of the 35 HB and 36 of the 41 dHB were successfully predicted by the OPLS-DA model with an accuracy of 88.2%.

In total, 61 metabolites were measured. To explore the metabolic differences between HB and dHB, we used the training set to build the OPLS-DA model. As shown in Figure 2A, the score plots of the OPLS-DA model showed that the dHB were obviously separated from HB with little overlap (R^2^X=0.38, R^2^Y=0.69, Q^2^=0.50), implying robust metabolic differences between HB and dHB. Moreover, as shown in Figure 2B, the results of permutation test suggested that the built OPLS-DA model was valid and not over-fitted. Meanwhile, the testing set was used to independently validate the reliability of the built model. As shown in Figure 3, the T-predicted scatter plot from the built OPLS-DA model showed that 31 of the 35 HB and 36 of the 41 dHB were correctly predicted, yielding a predictive accuracy of 88.2%. These results demonstrated that this OPLS-DA model built with urinary metabolites could be a potential method for objectively diagnosing dHB. Moreover, we found that samples from non-medicated patients showed a similar metabolic phenotype to samples from medicated patients in both groups (see Supplementary file 1).

### Differential urinary metabolites

**Figure 4:**
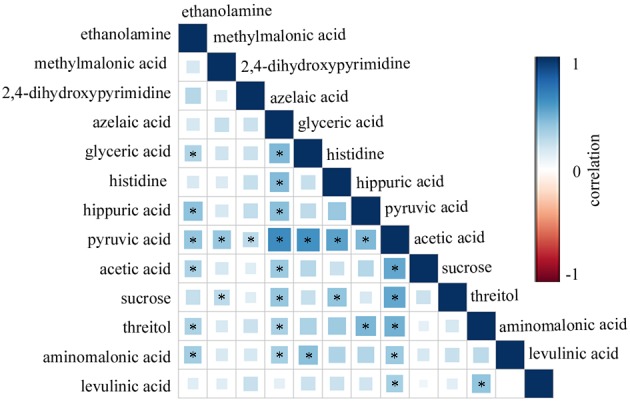
Pearson correlation analysis of differential urinary metabolites using R software.

By analyzing the OPLS-DA loadings plot, we found 13 differential urinary metabolites (VIP>1.0) responsible for discriminating dHB from HB (Table 2). As compared to the HB, the dHB were characterized by higher levels of azelaic acid, glyceric acid, histidine, hippuric acid, pyruvic acid, acetic acid, sucrose, threitol, aminomalonic acid and levulinic acid, along with lower levels of ethanolamine, methylmalonic acid and 2,4-dihydroxypyrimidine. These differential urinary metabolites belonged to Lipid metabolism, Amino acid metabolism, Oxidative stress and Energy Metabolism. Meanwhile, to assess the correlations between the identified differential metabolites, the Pearson correlation coefficient was used. As shown in Figure 4, there were relatively moderate correlations between pyruvic acid and other metabolites.

**Table 2:** Differential urinary metabolites attributed to discriminating HB and dHB patients.

**Metabolite**	**VIP**	**P-value^a^**	**P-adjusted^c^**	**Fold change^c^**	**Category**
ethanolamine	2.67	0.24	0.45	-0.34	Lipid metabolisim
azelaic acid	1.32	1.81E-07	1.17E-06	0.98	Oxidative stress
glyceric acid	1.09	0.06	0.14	0.50	Lipid metabolisim
histidine	1.51	8.04E-07	2.6E-06	0.96	Amino acid metabolism
methylmalonic acid	1.72	0.57	0.62	-0.11	Amino acid metabolism
hippuric acid	1.20	0.27	0.45	0.32	Amino acid metabolism
2,4-dihydroxypyrimidine	1.69	0.51	0.66	-0.09	Amino acid metabolism
pyruvic acid	1.06	0.0002	0.0006	0.34	Energy Metabolism
acetic acid	1.40	0.31	0.45	0.20	Lipid metabolisim
sucrose	1.24	0.86	0.86	0.11	Energy Metabolism
threitol	1.72	3.51E-07	1.5E-06	1.03	Lipid metabolisim
aminomalonic acid	1.28	0.55	0.65	0.32	Amino acid metabolism
levulinic acid	1.94	1.05E-07	1.35E-06	1.09	Amino acid metabolism

^a^P-values were derived from non-parametric Mann-Whitney U test. ^b^P-values were corrected using Benjamini and Hochberg False Discovery Rate method. ^c^Positive and negative values indicate higher and lower levels, respectively, in HB patients with depression.

### Potential biomarker panel

**Figure 5:**
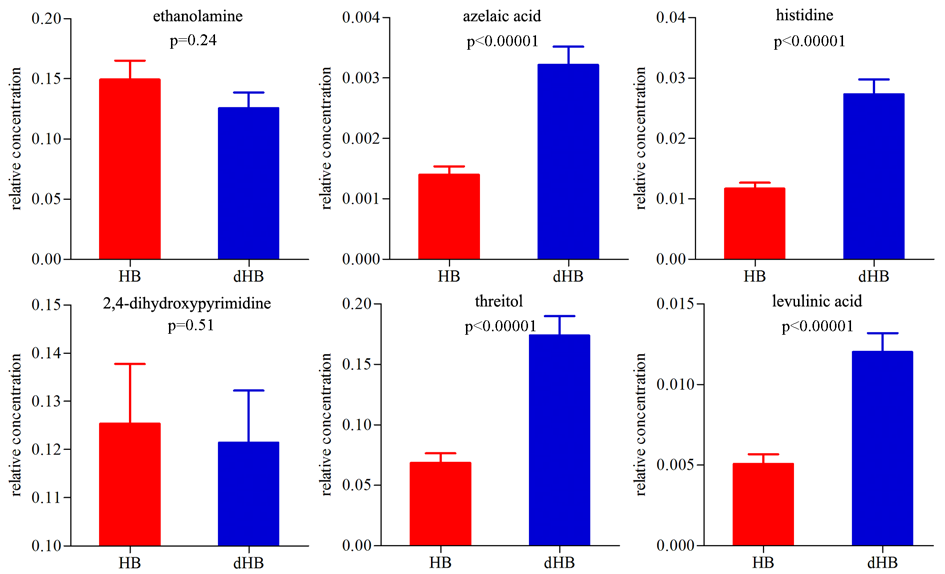
Relative concentrations of these six urinary metabolite biomarkers for dHB.

It is not economical and convenient to simultaneously measure 13 metabolites to diagnose dHB. Therefore, these differential metabolites were used as variables to further conduct step-wise logistic regression analysis. We used the BIC rule to determine the minimum number of metabolites in the potential biomarker panel. The results showed that six metabolites (ethanolamine, azelaic acid, histidine, threitol, 2,4-dihydroxypyrimidine and levulinic acid) could describe the most significant deviations between dHB from HB. The relative concentration of these six metabolites was displayed in Figure 5. The non-parametric Mann-Whitney U test was used to explore the group differences on these metabolites. The panel consisting of these six metabolites could yield acceptably high accuracies of 94.6% and 86.8% in the training and testing set, respectively. The panel consisting of these six metabolites had the similar accuracy to the panel consisting of 13 differential metabolites. The discriminative model was: P(Y=1) = 1/(1+e-y); y= -40.213*ethanolamine + 1518.415*azelaicacid + 303.788*histidine - 15.894*2,4-dihydroxypyrimidine + 28.621*threitol + 543.669*levulinic acid - 8.966. This model could be used to calculate the probability of depression in each HBV-infected patient.

### Diagnostic performance

**Figure 6:**
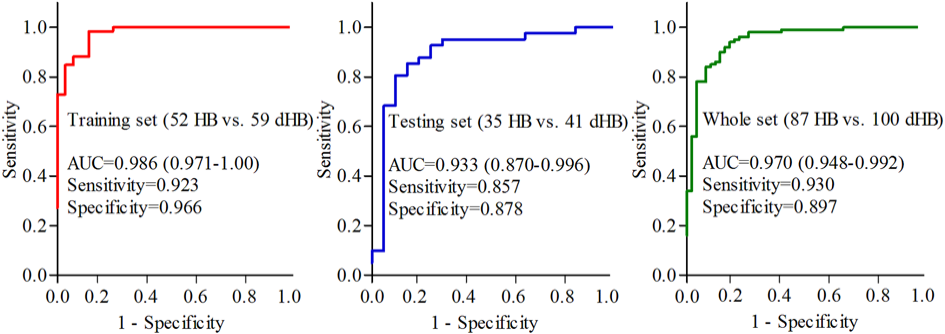
Diagnostic performance of the identified biomarker panel in training, testing and whole sets.

The ROC curve analysis was used to assess the diagnostic performance of this simplified panel. The areas under the ROC curve (AUC) was calculated to estimate how well dHB would be discriminated from HB (discriminating power). An AUC of 1.0 represented a perfect discrimination, and 0.5 referred to a case with no discrimination at all. The results showed that this simplified panel could effectively discriminate dHB from HB with an AUC of 0.986 (95% confidence interval (CI) = 0.971-1.00) in the training set (Figure 6). Furthermore, this simplified panel could classify blinded samples from the testing set with an AUC of 0.933 (95%CI=0.870-0.996) (Figure 6). Given the biological reproducibility observed in both sets, we used all samples to conduct ROC analysis to increase the statistical power. The AUC was 0.970 (95%CI=0.948-0.992) in the whole set (Figure 6). These results showed that the diagnostic performance was similar between this simplified panel and the OPLS-DA model built with all the differential metabolites, suggesting the efficacy of this simplified panel in dHB detection. Meanwhile, we found that these six identified biomarkers had no sex specificity (see Supplementary file 1).

### Subsequent analysis

**Figure 7:**
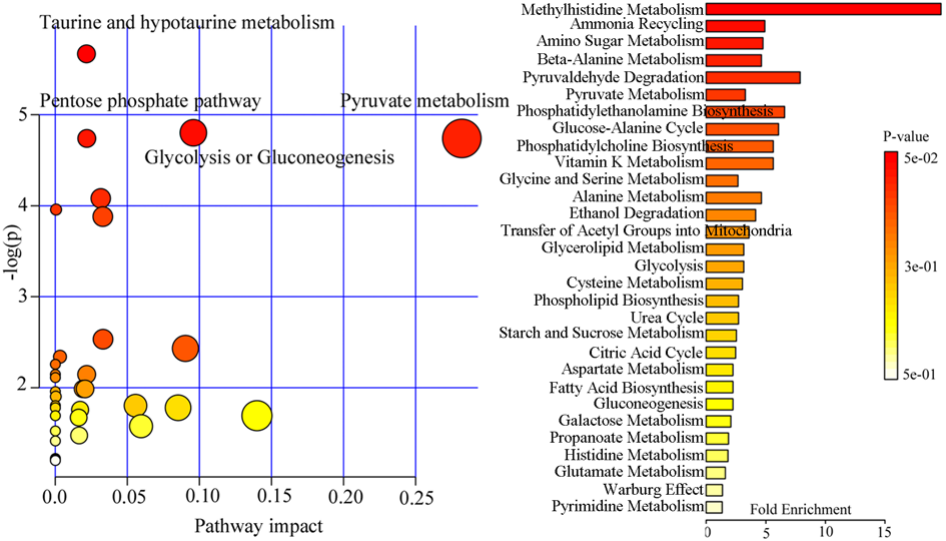
Affected metabolic pathways and functional enrichment analysis using 13 differential metabolites.

The 13 differential metabolites were imported into MetaboAnalyst 3.0 to conduct pathway analysis and functional enrichment analysis. The pathway analysis showed that these metabolites were mainly involved in four metabolic pathways: (p-value<0.01, impact>0): Taurine and hypotaurine metabolism (p=0.004, impact=0.021); Glycolysis or Gluconeogenesis (p=0.009, impact=0.096); Pentose phosphate pathway (p=0.010, impact=0.022); Pyruvate metabolism (p=0.010, impact=0.282) (Figure 7A). The functional enrichment analysis showed that the top 3 infected functions were methylhistidine metabolism, ammonia recycling and amino sugar metabolism (Figure 7B).

## DISCUSSION

In this work, we used GC-MS-based metabolomics approach to further study the metabolic changes between dHB and HB. The OPLS-DA model showed that the dHB group could be obviously separated from the HB group. In total, we identified 13 differential urinary metabolites (VIP>1.0) responsible for the discrimination. These differential urinary metabolites belonged mainly to Lipid metabolism and Amino acid metabolism. Further analysis showed that six of the 13 differential metabolites (ethanolamine, azelaic acid, histidine, threitol, 2,4-dihydroxypyrimidine and levulinic acid) were defined as candidate diagnostic biomarkers for dHB. The panel consisting of these six metabolites could yield an AUC of 0.986 in the training set and 0.933 in the testing set. Our findings further indicated that the urinary metabolite biomarkers could aid in the future development of objective diagnostic methods for dHB.

The levels of several differential metabolites were not found to be significantly changed (p-value<0.05) by the non-parametric Mann-Whitney U test (univariate analysis). However, the OPLS-DA model (multivariate analysis) still viewed these metabolites as differential metabolites responsible for the discrimination between the two groups. Previous metabolic studies also reported the similar results [27,28]. This was because the multivariate analysis found the highest discrimination power after adding these metabolites into the discrimination model. These results demonstrated that the multivariate analysis had some advantages over univariate analysis in detecting the potential significance of subtle metabolic changes between the different groups [29].

Metabolomics has been extensively applied to capture the metabolic changes of diseases [30]. The metabolic phenotypes might be different in different disease states. Using GC-MS-based metabolomics approach to compare the urinary metabolic phenotypes of depressed patients and healthy controls, a previous study identified six potential urinary biomarkers (sorbitol, uric acid, azelaic acid, quinolinic acid, hippuric acid, and tyrosine) for diagnosing depression [31]. In this study, the azelaic acid was also identified as biomarker for diagnosing dHB, and the hippuric acid was identified as differential metabolites. The other four metabolites were not identified as differential metabolites responsible for the discrimination between dHB and HB. Meanwhile, we used these six biomarkers to diagnose dHB in this study, and found that the diagnostic accuracy of these six biomarkers were inferior to the diagnostic accuracy of our panel (72.15% vs. 94.6%). These results suggested that the metabolic phenotypes were different between patients with only depression and dHB. Therefore, it might be inappropriate to use the previous panel to diagnosing depression in HBV-infected patients [31].

Previous study found that the decreased central energy production in depressed patients might be mirrored by the peripheral metabolic perturbations [27]. The significantly decreased level of plasma pyruvic acid (energy metabolism) was found in depressed patients [32]. Here, we found that there were relatively moderate correlations between pyruvic acid and other differential metabolites, and the Pyruvate metabolism was affected in dHB. HBV was frequently accompanied by many complications, such as hepatocellular carcinoma. Moreover, HBV was responsible for over half of hepatocellular carcinoma cases worldwide [33]. Another study reported that the pyruvic acid could be used for lipid synthesis, which was important for tumor cell proliferation and survival [34]. In addition, researchers found the correlation between lipid synthesis and glycolysis [35]. In this study, we found that the glycolysis was affected in dHB. Meanwhile, the Taurine and hypotaurine metabolism was found to be affected in dHB. And, the functional enrichment analysis found that some related functions of amino acid metabolism, such as methylhistidine metabolism, ammonia recycling and amino sugar metabolism, were affected in dHB. Taken together, these results indicated that the lipid metabolism and amino acid metabolism might be disturbed in depressed HBV-infected patients.

Several limitations existed in this study: 1) the number of recruited patients in both training set and testing set was relatively small; future large-scale studies were needed to validate our conclusions; 2) all patients were from the same place; then there might be ethno- and site-specific biases; Future studies with heterogeneous populations were required to conduct across different sites; 3) the diagnostic performance of this panel was confirmed solely by discriminating dHB from HB; Future work should study whether or not these biomarkers could be used to differentiate dHB from HBV-infected patients with other psychiatric disorders; 4) previous metabolic study reported that the medications might have a non-significant effect on the urinary metabolites [36]. Here, we also found the similar results. However, further studies should collect larger samples to determinate the potential influences of drugs on the urinary metabolites; 5) only urinary metabolites were studied here, future studies should collect other biological samples from patients, such as plasma and cerebrospinal fluid, to check whether these urinary biomarkers are physiologically relevant to disease pathogenesis and related to brain functionality; 6) we did not address whether the metabolic changes in urine was a consequence or a cause of depression in HBV-infected patients, which was needed future studies to find out; 7) except for well-matched demographic data, such as gender, age and BMI in the included patients, future studies should also consider the other possible confounding factors, such as smoking status, lifestyle and alcohol consumption; 8) there was lack of imaging and known assays for depression; 9) the number of differential metabolites might be not enough to obtain the robust results of pathway analysis and enrichment analysis, thus future studies were still needed to validate and support these results.

In conclusion, using GC-MS-based metabolomics approach to study the metabolic phenotypes of dHB, 13 differential metabolites responsible for separating dHB from HB were identified, which mainly belonged to Lipid metabolism and Amino acid metabolism. Moreover, a panel consisting of six differential metabolites (ethanolamine, azelaic acid, histidine, threitol, 2,4-dihydroxypyrimidine and levulinic acid) was identified, which displayed a good diagnostic performance (yielding an AUC of 0.986 in the training set and 0.933 in the testing set).

## MATERIALS AND METHODS

### Patients recruitment

This study was reviewed and approved by the Ethical Committee of Xinxiang Medical University, and the methods were strictly performed according to the approved guidelines and regulations. The dHB were included in this study if they met the following criteria: i) depression was diagnosed in a Structured Psychiatric Interview using DSM-IV-TR criteria; ii) the 17-item Hamilton Depression Rating Scale (HDRS) score, which was used to assess the symptom severity, was more than 17; iii) patients did not receive any antidepressant medications; iv) cirrhosis patients co-infected with human immunodeficiency virus or hepatitis C virus were not included; v) patients with other psychiatric disorders or any pre-existing bodily disorders were not included; vi) patients with alcohol or illicit drug problems were not included; vii) patients older than 40 years of age (middle or old age) were included; and viii) pregnant women were also excluded. The HB met the last six criteria were recruited. All patients were recruited from the First Affiliated Hospital of Xinxiang Medical University, and provided the written informed consent before sample collection.

### GC-MS acquisition

First, the included patients used the sterile cup to collect the morning urine samples (9am to 10am). Then, after quickly transferring the urine samples into the sterile tube, we sent the urine samples to our lab under low-temperature condition. After centrifugation (1500g x 10 minutes), the resulted supernatant was immediately divided into equal aliquots and stored at -80 °C for subsequent analysis. Prior to GC-MS analysis, the following steps were completed: 1) mixed and vortexed 15μl urine and 10°C L-leucine-13C6 (0.02 mg/ml, internal standard solution); 2) added 15μl urease into the mixed solution to degrade the urea at 37 °C for 60 minutes; 3) extracted the mixture successively using 240I1/4l and 80I1/4l of ice-cold methanol; 4) vortexed the obtained mixture for 30 seconds and then conducted centrifugation (at 4 °C, 14000 rpm x5 minutes); 5) at room temperature, transferred 224ul supernatant into a glass vial for vacuum drying; 6) derivatized the obtained dried metabolic extract using 30μl methoxyamine (20 mg/ml) at 37 °C for 90 minutes; 7) added 30μl of N,O-bis(trimethylsilyl) trifluoroacetamide with 1% trimethylchlorosilane into the obtained dried metabolic extract; 8) heated the mixture at 70 °C for 60 minutes to obtain trimethylsilyl derivatives; 9) after derivatization and cooling to room temperature, added 1.0μl derivative into the GC-MS system. Meanwhile, the detailed information of quality control samples preparation was described in Supplementary file 1.

### GC-MS analysis

The GC-MS analysis was performed mainly referring to the previous study [21]. Briefly, we injected 1 I1/4L derivative solution into the Agilent 7980 GC system (Agilent Technologies Inc., USA). Then, we used the HP-5 MS fused silica capillary column (30 m A- 0.25 mm A- 0.25 I1/4m, Agilent, USA) to do separation. The helium carrier gas was set a flow rate of 1 mL/minute. We set the injector temperature at 280 °C; the column temperature was initially set at 80 °C (held for two minutes) and then equably (10 °C/minute) increased to 320 °C (held for six minutes). Subsequently, we introduced the column effluent into the ion source of an Agilent 5975 mass selective detector (Agilent Technologies Inc., USA). We set the MS quadrupole temperature at 150 °C and the ion source temperature at 230 °C. Data acquisition was performed in the full scan mode from m/z 50 to 550. After the data was conversion into a NetCdf file format, we used TagFinder to process the GC-MS metabolite profiles [22]. This processing used deconvolution, alignment and data reduction to obtain a list of mass and retention time pairs with corresponding intensities for all detected peaks from each data file in the data set. The produced three-dimensional data set, including peak index (RT-m/z pair) and normalized peak area percentages, was imported into SIMCA-P 13.0 (Umetrics, Umeå, Sweden) for further analysis.

### Metabolomic data analysis

If a variable had a nonzero measurement value in at least 80% of the variables within one of the two subsets, the variable was included in the data set; otherwise the variable was removed [23]. The levels of these eligible identified metabolites were normalized to creatinine, and then imported into the SIMCA-P 13.0 (Umetrics, Umeå, Sweden) to conduct metabolomic data analysis. The orthogonal partial least squares discriminant analysis (OPLS-DA) was used to visualize the discrimination between dHB and HB [24]. The three parameters (R^2^X, R^2^Y and Q^2^Y) were used to assess the quality of the built OPLS-DA model [24]. The former two parameters and last parameter were used to assess the goodness-of-fit and predictability of the model, respectively [24]. Meanwhile, a 300-iteration permutation test was further conducted to investigate whether the built model was over-fitted or not. If the values of R^2^ and Q^2^ from the original model were higher than their corresponding values from the permutation test, the built model was deemed valid and not over-fitted [24]. By analyzing the OPLS-DA loadings plot, the metabolites with variable importance plot (VIP) > 1 (equivalent to a p-value<0.05) were viewed as the differential urinary metabolites contributing to sample discrimination [24].

### Statistical analysis

The SPSS 19.0 was used to conduct the subsequent analysis. To obtain a simplified potential biomarker panel, the identified differential metabolites were used to conduct step-wise logistic regression analysis based on Bayesian Information Criterion (BIC). The receiver-operating characteristic (ROC) curve analysis was used to assess the diagnostic performance of this identified panel. The students t-test, nonparametric Mann-Whitney U test and Chi-square test were performed when appropriate. The p-values were corrected using Benjamini and Hochberg False Discovery Rate method [25]. The pearson correlation analysis was performed in R software, using the corrplot package. The correlation analysis was used to assess the correlations between the metabolites. The online software MetaboAnalyst 3.0 was used to conduct pathway analysis and functional enrichment analysis [26]. All tests were two-sided, and a p-value<0.05 was considered to be statistically significant.

## SUPPLEMENTARY MATERIAL

Supplementary file 1

Data set

## References

[1] Cooksley WG, Piratvisuth T, Lee SD, Mahachai V, Chao YC, Tanwandee T, Chutaputti A, Chang WY, Zahm FE, Pluck N. Peginterferon alpha-2a (40 kDa): an advance in the treatment of hepatitis B e antigen-positive chronic hepatitis B. J Viral Hepat. 2003; 10:298-305. 10.1046/j.1365-2893.2003.00450.x 12823597

[2] Cornberg M, Jaroszewicz J, Manns MP, Wedemeyer H. Treatment of chronic hepatitis B. Minerva Gastroenterol Dietol. 2010; 56:451-65. 21139543

[3] Zhu HP, Gu YR, Zhang GL, Su YJ, Wang KE, Zheng YB, Gao ZL. Depression in patients with chronic hepatitis B and cirrhosis is closely associated with the severity of liver cirrhosis. Exp Ther Med. 2016; 12:405-09. 10.3892/etm.2016.3271 27347069 PMC4906933

[4] Luscher B, Shen Q, Sahir N. The GABAergic deficit hypothesis of major depressive disorder. Mol Psychiatry. 2011; 16:383-406. 10.1038/mp.2010.120 21079608 PMC3412149

[5] GBD 2015 Disease and Injury Incidence and Prevalence Collaborators. Global, regional, and national incidence, prevalence, and years lived with disability for 310 diseases and injuries, 1990-2015: a systematic analysis for the Global Burden of Disease Study 2015. Lancet. 2016; 388:1545-602. 10.1016/S0140-6736(16)31678-6 27733282 PMC5055577

[6] Chen JW, Xie SQ. Agomelatine versus paroxetine in treating depressive and anxiety symptoms in patients with chronic kidney disease. Neuropsychiatr Dis Treat. 2018; 14:547-52. 10.2147/NDT.S159636 29497298 PMC5818845

[7] Gallegos-Orozco JF, Fuentes AP, Gerardo Argueta J, PA(c)rez-Pruna C, Hinojosa-Becerril C, Sixtos-Alonso MS, Cruz-Castellanos S, GutiA(c)rrez-Reyes G, Olivera-MartA-nez MA, GutiA(c)rrez-Ruiz MC, Kershenobich D. Health-related quality of life and depression in patients with chronic hepatitis C. Arch Med Res. 2003; 34:124-29. 10.1016/S0188-4409(03)00003-1 12700008

[8] Raison CL, Broadwell SD, Borisov AS, Manatunga AK, Capuron L, Woolwine BJ, Jacobson IM, Nemeroff CB, Miller AH. Depressive symptoms and viral clearance in patients receiving interferon-alpha and ribavirin for hepatitis C. Brain Behav Immun. 2005; 19:23-27. 10.1016/j.bbi.2004.05.001 15581735

[9] Arvand J, Shafiabadi A, Falsafinejad MR, Naderi N. Depression in patients with chronic hepatitis B: an experience on individual solution- focused therapy. Gastroenterol Hepatol Bed Bench. 2012; 5:166-68. 24834219 PMC4017476

[10] Chen JJ, Zeng BH, Li WW, Zhou CJ, Fan SH, Cheng K, Zeng L, Zheng P, Fang L, Wei H, Xie P. Effects of gut microbiota on the microRNA and mRNA expression in the hippocampus of mice. Behav Brain Res. 2017; 322:34-41. 10.1016/j.bbr.2017.01.021 28093256

[11] Mata DA, Ramos MA, Bansal N, Khan R, Guille C, Di Angelantonio E, Sen S. Prevalence of depression and depressive symptoms among resident physicians: a systematic review and meta-analysis. JAMA. 2015; 314:2373-83. 10.1001/jama.2015.15845 26647259 PMC4866499

[12] Chen JJ, Zheng P, Liu YY, Zhong XG, Wang HY, Guo YJ, Xie P. Sex differences in gut microbiota in patients with major depressive disorder. Neuropsychiatr Dis Treat. 2018; 14:647-55. 10.2147/NDT.S159322 29520144 PMC5833751

[13] Mitchell AJ, Vaze A, Rao S. Clinical diagnosis of depression in primary care: a meta-analysis. Lancet. 2009; 374:609-19. 10.1016/S0140-6736(09)60879-5 19640579

[14] Yang J, Chen T, Sun L, Zhao Z, Qi X, Zhou K, Cao Y, Wang X, Qiu Y, Su M, Zhao A, Wang P, Yang P, et al. Potential metabolite markers of schizophrenia. Mol Psychiatry. 2013; 18:67-78. 10.1038/mp.2011.131 22024767 PMC3526727

[15] Zheng P, Chen JJ, Zhou CJ, Zeng L, Li KW, Sun L, Liu ML, Zhu D, Liang ZH, Xie P. Identification of sex-specific urinary biomarkers for major depressive disorder by combined application of NMR- and GC-MS-based metabonomics. Transl Psychiatry. 2016; 6:e955. 10.1038/tp.2016.188 27845778 PMC5314113

[16] Hou LJ, Duan SP, Wang HW, Li WW, Song XW, Shen BS. Diagnosis of major depression in hepatitis B patients by urine metabolomics. Journal of Shanghai Jiaotong University. 2014; 34:1647-51.

[17] Hou LJ, Wang HW, Wei XX, Duan SP, Zhuo Y, Song XW, Shen BS. Urinary metabonomics for diagnosis of depression in hepatitis B virus-infected patients. Iran Red Crescent Med J. 2015; 17:e27359. 10.5812/ircmj.17(4)2015.27359 26023351 PMC4443390

[18] Williams R, Lenz EM, Wilson AJ, Granger J, Wilson ID, Major H, Stumpf C, Plumb R. A multi-analytical platform approach to the metabonomic analysis of plasma from normal and Zucker (fa/fa) obese rats. Mol Biosyst. 2006; 2:174-83. 10.1039/b516356k 16880935

[19] Chen JJ, Zhou CJ, Zheng P, Cheng K, Wang HY, Li J, Zeng L, Xie P. Differential urinary metabolites related with the severity of major depressive disorder. Behav Brain Res. 2017; 332:280-87. 10.1016/j.bbr.2017.06.012 28624318

[20] Xu XJ, Zheng P, Ren GP, Liu ML, Mu J, Guo J, Cao D, Liu Z, Meng HQ, Xie P. 2,4-Dihydroxypyrimidine is a potential urinary metabolite biomarker for diagnosing bipolar disorder. Mol Biosyst. 2014; 10:813-19. 10.1039/c3mb70614a 24457555

[21] Shao WH, Fan SH, Lei Y, Yao GE, Chen JJ, Zhou J, Xu HB, Liu HP, Wu B, Zheng P, Fang L, Xie P. Metabolomic identification of molecular changes associated with stress resilience in the chronic mild stress rat model of depression. Metabolomics. 2013; 9:433-43. 10.1007/s11306-012-0460-2

[22] Luedemann A, Strassburg K, Erban A, Kopka J. TagFinder for the quantitative analysis of gas chromatography--mass spectrometry (GC-MS)-based metabolite profiling experiments. Bioinformatics. 2008; 24:732-37. 10.1093/bioinformatics/btn023 18204057

[23] Bijlsma S, Bobeldijk I, Verheij ER, Ramaker R, Kochhar S, Macdonald IA, van Ommen B, Smilde AK. Large-scale human metabolomics studies: a strategy for data (pre-) processing and validation. Anal Chem. 2006; 78:567-74. 10.1021/ac051495j 16408941

[24] Mahadevan S, Shah SL, Marrie TJ, Slupsky CM. Analysis of metabolomic data using support vector machines. Anal Chem. 2008; 80:7562-70. 10.1021/ac800954c 18767870

[25] Benjamini Y. Discovering the false discovery rate. J R Stat Soc Ser A Stat Soc. 2010; 72:405-16. 10.1111/j.1467-9868.2010.00746.x

[26] Xia J, Sinelnikov IV, Han B, Wishart DS. MetaboAnalyst 3.0--making metabolomics more meaningful. Nucleic Acids Res. 2015; 43:W251-7. 10.1093/nar/gkv380 25897128 PMC4489235

[27] Zheng P, Wang Y, Chen L, Yang D, Meng H, Zhou D, Zhong J, Lei Y, Melgiri ND, Xie P. Identification and validation of urinary metabolite biomarkers for major depressive disorder. Mol Cell Proteomics. 2013; 12:207-14. 10.1074/mcp.M112.021816 23111923 PMC3536901

[28] Lin L, Chen XM, Liu RH. Novel urinary metabolite signature for diagnosing postpartum depression. Neuropsychiatr Dis Treat. 2017; 13:1263-70. 10.2147/NDT.S135190 28546751 PMC5436788

[29] MacIntyre DA, Jiménez B, Lewintre EJ, MartA-n CR, Schäfer H, Ballesteros CG, Mayans JR, Spraul M, GarcA-a-Conde J, Pineda-Lucena A. Serum metabolome analysis by 1H-NMR reveals differences between chronic lymphocytic leukaemia molecular subgroups. Leukemia. 2010; 24:788-97. 10.1038/leu.2009.295 20090781

[30] Kaddurah-Daouk R, Kristal BS, Weinshilboum RM. Metabolomics: a global biochemical approach to drug response and disease. Annu Rev Pharmacol Toxicol. 2008; 48:653-83. 10.1146/annurev.pharmtox.48.113006.094715 18184107

[31] Zheng P, Chen JJ, Huang T, Wang MJ, Wang Y, Dong MX, Huang YJ, Zhou LK, Xie P. A novel urinary metabolite signature for diagnosing major depressive disorder. J Proteome Res. 2013; 12:5904-11. 10.1021/pr400939q 24224655

[32] Zheng P, Gao HC, Li Q, Shao WH, Zhang ML, Cheng K, Yang DY, Fan SH, Chen L, Fang L, Xie P. Plasma metabonomics as a novel diagnostic approach for major depressive disorder. J Proteome Res. 2012; 11:1741-48. 10.1021/pr2010082 22239730

[33] Kessler RC, Berglund P, Demler O, Jin R, Koretz D, Merikangas KR, Rush AJ, Walters EE, Wang PS, and National Comorbidity Survey Replication. The epidemiology of major depressive disorder: results from the National Comorbidity Survey Replication (NCS-R). JAMA. 2003; 289:3095-105. 10.1001/jama.289.23.3095 12813115

[34] Gogvadze V, Orrenius S, Zhivotovsky B. Mitochondria in cancer cells: what is so special about them? Trends Cell Biol. 2008; 18:165-73. 10.1016/j.tcb.2008.01.006 18296052

[35] Yu Y, Shen H, Yu H, Zhong F, Zhang Y, Zhang C, Zhao J, Li H, Chen J, Liu Y, Yang P. Systematic proteomic analysis of human hepotacellular carcinoma cells reveals molecular pathways and networks involved in metastasis. Mol Biosyst. 2011; 7:1908-16. 10.1039/c0mb00265h 21468425

[36] Zheng P, Wei YD, Yao GE, Ren GP, Guo J, Zhou CJ, Zhong JJ, Cao D, Zhou LK, Xie P. Novel urinary biomarkers for diagnosing bipolar disorder. Metabolomics. 2013; 9:800-08. 10.1007/s11306-013-0508-y

