# Supplementary material for: GC-MS-based metabolomics approach to diagnose depression in hepatitis B virus-infected patients with middle or old age
Source: Aging (Albany NY). 2018 Sep 2;10(9):2252–65. doi: 10.18632/aging.101535 (PMC6188484; doi:10.18632/aging.101535)
Supplement: Supplementary file 1 [file aging-10-101535-s001.pdf]

SUPPLEMENTARY MATERIAL

Please browse the links in Full Text version of this manuscript to see Data Sets.

Quality control samples

The 15µl urine from each samples were pooled and fully mixed. After centrifugation (1500g x 10 minutes), the resulted supernatant was immediately divided into equal aliquots (15µl) and stored at -80°C for subsequent analysis. In total, there were 18 quality control (QC) samples. We run the pooled QC samples alongside the sample runs for controlling batch and unwanted sources of variations. Continuous analysis of the samples was performed in a random sequence. One QC sample was set after ten experimental samples, which was used to monitor and evaluate the stability of the system and the reliability of the experimental data. The detected values of the QC samples were extracted based on the data detected by the mass spectrometry and the RSD was calculated to remove the molecules with RSD > 30%. The QC samples running alongside samples as PCA plots were showed in Figure S1A. No QC samples was 2SD from the mean (Figure S1B). These results indicated the stability of the system and the reliability of the experimental data.

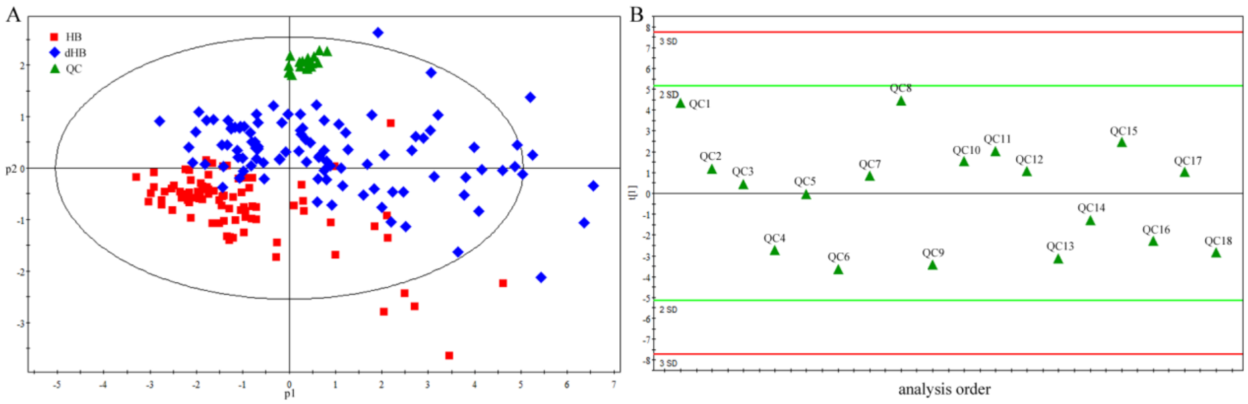

Figure S1. QC samples showed the stability of system and the reliability of the experimental data.

Medication effects on metabolites

All the included patients did not receive any antidepressant medications, but there were 11 patients in the HB group and 16 patients in the dHB group receiving medicines for treating HBV, such as telbivudine, lamivudine, interferon and adefovir dipivoxil. Previous metabolic study reported that the medications might have a non-significant effect on the urinary metabolites [1]. In this study, the OPLS-DA model built with non-medicated and medicated patients in HB group

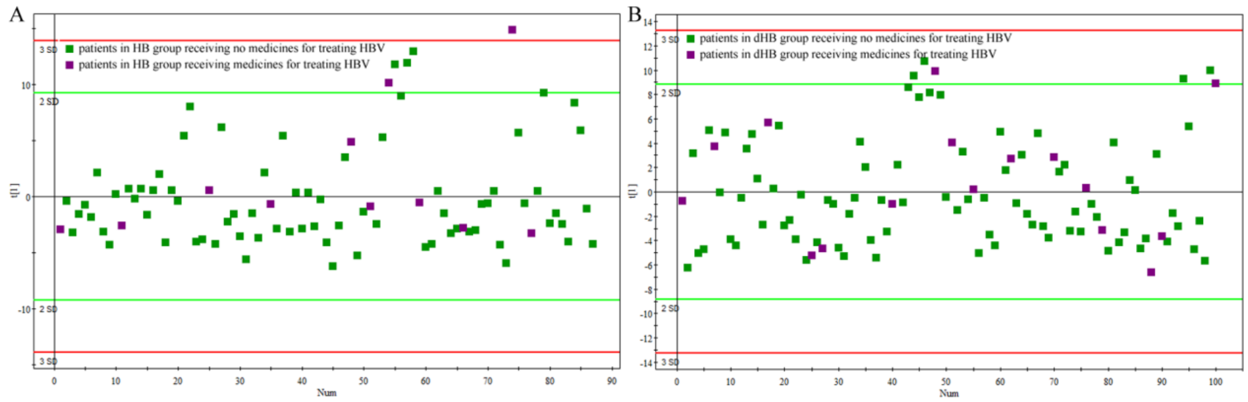

Figure S2. Non-medicated and medicated patients had similar metabolic phenotype in both groups.

showed that the non-medicated and medicated patients could not be well separated, which demonstrated that the non-medicated and medicated patients in HB group had the similar metabolic phenotype (Figure S2A). Meanwhile, the OPLS-DA model built with non-medicated and medicated patients in dHB group also showed the similar results (Figure S2B). These results indicated that the medicines for treating HBV might have little effect on the urinary metabolites. Limited by the small number of medicated samples, further studies should collect larger samples to determinate the potential influences of drugs on the urinary metabolites.

Sex-differences analysis

In order to check the sex-differences of urinary metabolites, we used the female and male patients in the HB group to build the OPLS-DA model (Figure S4A). The results showed that the female and male patients could not be well separated, which demonstrated that the female and male HB had the similar metabolic phenotype. Meanwhile, the OPLS-DA model built with female and male patients in the dHB group also showed the similar results (Figure S4B). Meanwhile, in this study, we found that these identified biomarkers had no sex specificity. The panel consisting of these biomarkers could effectively distinguish female dHB from female HB with AUC of 0.985 (95% confidence interval (CI): 0.968-1.00) (Figure S4A), and male dHB from male HB with AUC of 0.956 (95%CI: 0.914-0.998) (Figure S4B). These results demonstrated that there were no sex-differences in urinary metabolites in both HB and dHB groups.

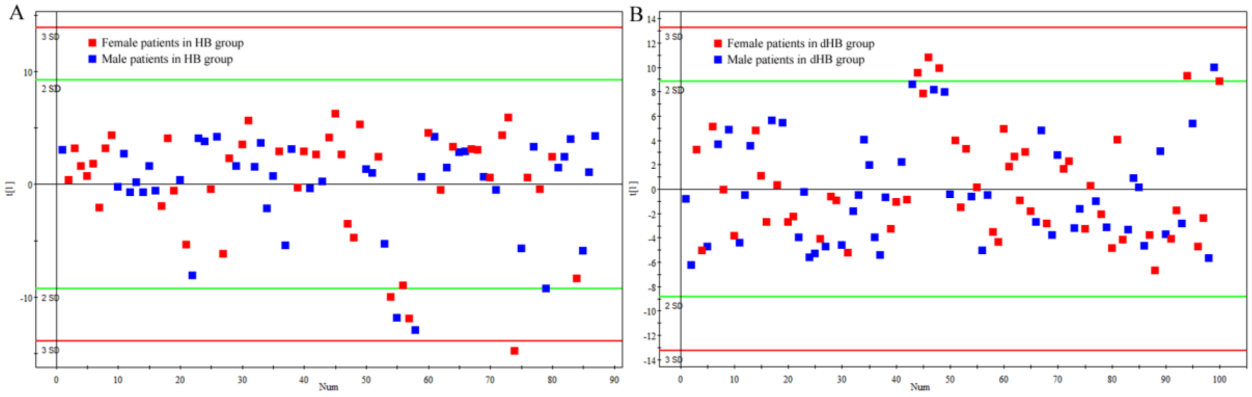

Figure S3. Female and male patients in both group had the similar metabolic phenotype.

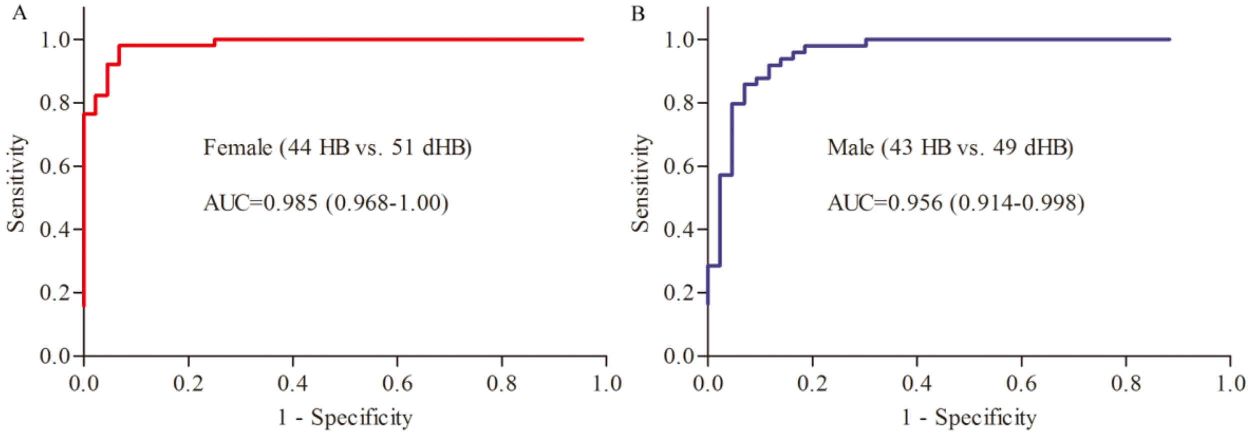

Figure S4. Diagnostic performances of the panel in diagnosing female and male dHB.

REFERENCES

1. Zheng P, Wei YD, Yao GE, Ren GP, Guo J, Zhou CJ, Zhong JJ, Cao D, Zhou LK, Xie P. Novel urinary biomarkers for diagnosing bipolar disorder. Metabolomics. 2013; 9: 800-8. https://doi.org/10.1007/s11306-013-0508-y
